# The gut metabolite arachidonic acid alleviates intestinal injury induced by a *Toxoplasma gondii* strain isolated from a wild rodent

**DOI:** 10.1186/s13071-026-07389-y

**Published:** 2026-05-02

**Authors:** Hao Yuan, Yining Song, Linchong Nie, Zipeng Yang, Liulu Yang, Kunmei Yang, Yurong Yang, Wenhao Li, Xianghe Wang, Xiu-Xiang Zhang, Yan Hua, Zi-Guo Yuan

**Affiliations:** 1 Guangdong Provincial Key Laboratory of Zoonosis Prevention and Control, College of Veterinary Medicine, South China Agricultural University, Guangzhou, China; 2 Laboratory of Veterinary Pathology, College of Veterinary Medicine, Henan Agricultural University, Zhengzhou, 450002 China; 3 College of Agriculture, South China Agricultural University, Guangzhou, China; 4 Guangdong Provincial Key Laboratory of Silviculture, Protection and Utilization, Guangdong Academy of Forestry, Guangzhou, 510520 China

**Keywords:** *Toxoplasma gondii*, Gut metabolite, Arachidonic acid

## Abstract

**Background:**

Wild isolates of *Toxoplasma gondii* may exhibit different virulence characteristics and host adaptability compared with those of laboratory strains. In this study, we isolated a novel rodent-derived *T. gondii* strain, denoted TgRodGz1, and evaluated its pathogenic features.

**Methods:**

TgRodGz1 was isolated from T. gondii-positive wild rodents in Guangdong Province and compared with the RH and Me49 strains in C57BL/6 mice. Virulence and intestinal injury were evaluated by survival analysis, brain cyst quantification, histopathology, tight junction assessment and qPCR. Gut microbiota and metabolic alterations were analyzed by metagenomic sequencing and LC–MS/MS-based metabolomics.

**Results:**

Compared with the*T. gondii* laboratory strains RH and Me49, TgRodGz1 was associated with more pronounced intestinal injury, including villus atrophy, barrier disruption and downregulation of tight junction proteins and increased gut permeability and inflammation. Metagenomic analysis revealed significant intestinal flora dysbiosis, with a marked reduction in beneficial bacteria and expansion of pathogenic bacteria. Metabolomic analysis revealed suppression of arachidonic acid (ARA) metabolism during TgRodGz1 infection. Supplementation with ARA did not directly inhibit parasite growth but significantly alleviated intestinal lesions, reduced brain cyst burden and attenuated inflammatory responses, including microglial activation.

**Conclusions:**

These findings suggest that TgRodGz1 represents a distinct *T. gondii* genotype associated with pronounced intestinal pathology and suggest that ARA supplementation may alleviate intestinal and neuroinflammatory changes associated with *T. gondii* infection.

**Graphical Abstract:**

**Figure:**
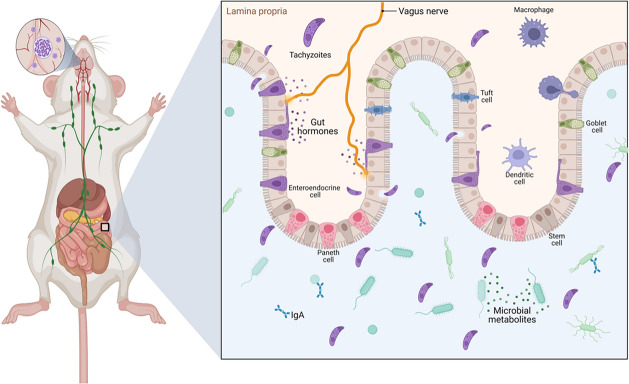

**Supplementary Information:**

The online version contains supplementary material available at 10.1186/s13071-026-07389-y.

## Background

Toxoplasma gondii（*T. gondii*）is a protozoan parasite capable of infecting a wide range of warm-blooded animals, including humans and diverse wildlife species such as the Chinese crocodile lizard (*Shinisaurus crocodilurus*). Due to its complex life-cycle and broad host range, *T. gondii* serves as an important model for studying parasite evolution and host adaptation [1]. *T. gondii* exhibits significant genetic diversity, particularly in strains isolated from the wild, which reflects its long-term evolutionary adaptation to various ecological niches [2, 3]. However, traditional studies have mainly focused on laboratory strains (e.g. Me49 and RH) which, due to long-term in vitro passage, have relatively simple genetic backgrounds and may have—at least partially—lost their natural virulence. In contrast, wild isolates directly derived from natural hosts may retain stronger environmental adaptability and pathogenicity, showing in particular specific tissue and organ tropism driven by genetic evolution [4, 5].

From the genetic evolution perspective, the virulence and host specificity of *T. gondii* strains are closely correlated with the polymorphism of their genomes [6]. Multi-locus genotyping studies have demonstrated that *T. gondii* encompasses multiple lineages (Type I, II, III and atypical strains) [6]. Wild *T. gondii* isolates, like TgRodGz1, a novel strain isolated from wild rodents, are subject to natural selection, often carry a higher proportion of virulence effector proteins (e.g. rhoptry proteins [ROPs], Dense Granule proteins [GRAs]) and demonstrate distinctive pathogenic features [7, 8]. In wild hosts, the high mortality rate resulting from predation risk prompts *T. gondii* to accelerate propagation through rapid proliferation (up-regulating the activity of AP2 transcription factors); the nutritional constraints of the intestinal microenvironment drive gene mutations in the major immunogene complex (MIC), enhancing the targeting of intestinal epithelium [9–11]. This "genetic diversity–ecological pressure–phenotypic adaptation" co-evolution drives the virulence differentiation and tissue and organ tropism of *T. gondii* strains.

The intestine serves as a critical target organ for *T. gondii* infection. During the acute infection phase, *T. gondii* oocysts enter the host's small intestine via oral ingestion, invade intestinal epithelial cells and undergo schizogony to produce tachyzoites [12]. These tachyzoites disrupt the intestinal mucosal structure and trigger acute inflammatory responses [13]. Research has demonstrated that *T. gondii* infection results in the down-regulation of tight junction proteins such as Occludin and ZO-1 in intestinal epithelial cells, thereby compromising the intestinal barrier integrity and increasing permeability, which facilitates parasite survival and transmission [13].

The host immune response exhibits a dual role in *T. gondii*-induced intestinal injury. Specifically, the innate immune system detects the profilin protein of *T. gondii* via Toll-like receptor 11 (TLR11), subsequently activating the MyD88-dependent signaling pathway and promoting the production of interleukin-12 ( IL-12) and interferon-gamma (IFN-γ), which effectively restricts parasite dissemination [14, 15]. An exaggerated type 1 T helper (Th1) cell immune response may lead to excessive inflammation and subsequent damage to intestinal tissues [16].

Infection with *T. gondii* activates the host's immune response and induces intestinal microbiota dysbiosis. During the early phase of infection, beneficial bacteria such as *Lactobacillus* and *Bifidobacterium* decrease in abundance, while *Escherichia coli* and other potential pathogens proliferate abnormally. These changes contribute to intestinal inflammation [17, 18]. This imbalance in the intestinal flora can also affect the systemic immune status through the gut–immune axis and amplify T-cell-mediated inflammatory responses [19]. In recent years, the use of probiotics and their metabolites in therapeutic strategies has attracted attention. Studies show that oral administration of *Lactobacillus rhamnosus*, alpha-linolenic acid and other probiotics can alleviate intestinal tissue damage in mice infected with *T. gondii*, inhibit the expression of pro-inflammatory cytokines, restore the integrity of the mucosal barrier and improve the pathological state of the host [15, 18, 20].

In the study reported here, we isolated a novel atypical *T. gondii* strain (TgRodGz1) from wild rodents. The virulence and pathogenicity of TgRodGz1 were compared with those of RH and Me49, standard *T. gondii* laboratory strains. Our results demonstrated that TgRodGz1 infection led to more severe intestinal damage, demonstration a pathogenicity greater than that of the RH and Me49 strains. This enhanced pathogenicity was closely associated with dysregulation of the intestinal microbiota. By combining metagenomic sequencing and metabolomics analyses, we found that TgRodGz1 infection induced a significant reduction in beneficial bacteria and an expansion of harmful bacteria, compared to the two standard strains. Additionally, metabolomics analysis revealed a decrease in the level of arachidonic acid (ARA) in the intestine during TgRodGz1 infection. To further explore the role of ARA in the infection process, an ARA supplementation mice model was established. Our experimental results indicated that oral ARA supplementation alleviated *T. gondii*-induced intestinal inflammation and mitigated the imbalance in the intestinal microbiota.

In conclusion, this study isolated a new atypical *T. gondii* strain with enhanced pathogenicity and identified the mechanism through which this strain promotes intestinal inflammation by regulating intestinal microbiota and metabolites.

## Methods

### Experimental animals and *T. gondii* strains

Five-week-old male C57BL/6 mice were purchased from Guangzhou Southern Medical University Laboratory Animal Science and Technology Development Co., Ltd. (both Guangzhou, China) The *T. gondii* Me49 and RH strains and human foreskin fibroblasts (HFFs) were maintained in the Parasite Laboratory of the College of Veterinary Medicine, South China Agricultural University (Guangzhou, China). The *T. gondii* TgRodGz1 strain was isolated from* T. gondii*-positive tissue samples of wild rodents during the sample collection period shown in Additional file 1: Table S1. All experimental animals were provided with sufficient food and water throughout the experiment. For all analyses, all animals were anesthetized with 6% isoflurane inhalation (MedChemExpress, Monmouth Junction, NJ, USA; catalog no. HY-A0134), followed by euthanasia by cervical dislocation. All animal experiments were approved by the Experimental Animal Ethics Committee of South China Agricultural University (Ethics No. 2024F306). All experiments adhered to the ARRIVE (Animal Research: Reporting of In Vivo Experiments) guidelines.

### Detection of *T. gondii* antibodies in serum

Serum samples from wild rodents were diluted at 1:25 and 1:200 and tested for *T. gondii* antibodies using the modified agglutination test (MAT). MAT was performed using a commercial kit (Toxo-Screen DA®; bioMérieux, Marcy-l'Étoile, France) according to the manufacturer’s instructions. Samples with MAT titers of ≥ 1:25 were considered to be positive for *T. gondii* antibodies.

### Research period

The collection of wild rodents was conducted between 8 May 2024 and 17 August 2024. The TgRodGz1 strain was isolated on 18 June 2024. The sequencing of the metagenome and metabolome of the mouse intestinal microbiota was conducted on 10 August 2024. The data analysis was conducted on 5 December 2024.

### Inoculation of mice with *T. gondii*-positive tissues

Tissue samples (each 50 g) from *T. gondii*-positive animals (heart, liver, spleen, lung, kidney and brain) were ground and each sample added to 250 ml of 0.85% sodium chloride solution, followed by digestion with pepsin solution. After digestion in a shaking water bath at 37 °C for 1 h, the process was terminated by the addition of an appropriate amount of Na_2_HCO_3_. The precipitate was then collected by centrifugation at 1000 *g* for 10 min and used to enrich *T. gondii* bodies. The digestive or grinding solution was inoculated into ten C57BL/6 mice, and body weight and clinical symptoms were recorded daily.

Following the method of Dubey [21], blood samples were collected from surviving mice inoculated with *T. gondii*-positive tissues at 30 days post-inoculation (DPI). Serum samples were diluted at ratios of 1:25 and 1:200 for the detection of *T. gondii* antibodies using MAT. At 60 DPI, the mice were euthanized and examined for cysts in their brains. If the mice were serologically positive but no *T. gondii* tachyzoites were detected, their tissue samples were inoculated into 10 C57BL/6 mice, and the above procedures were repeated.

The procedures for mice inoculated with tissue samples were as follows: for mice that died or had been euthanized, tissue samples were collected to prepare smears and then tested for the presence of *T. gondii* tachyzoites or cysts. If no cysts or tachyzoites were found, tissues from the lung, brain, heart, spleen, lymph nodes and thigh muscle were ground and inoculated into new mice to further test for infection.

### Gradient inoculation of *T. gondii* tachyzoites and observation and handling of mice

After grinding and mixing tissues and organs from mice infected with *T. gondii*, including the brain, lung, and mesenteric lymph nodes, the mixture was inoculated into HFF cell culture flasks under good growth conditions and placed in an incubator for culture to propagate the parasites and obtain viable, actively replicating tachyzoites. This step also helps remove host tissue debris and other contaminants present in the original tissue homogenate, thereby allowing the preparation of a cleaner and more standardized parasite inoculum for subsequent mouse infection experiments.

The culture medium was changed every 3–4 days, and the growth of *T. gondii* tachyzoites was observed and relevant data recorded. The number of tachyzoites in the tachyzoite-enriched culture medium was counted, and when the number of tachyzoites reached 10^7^/ml, a gradient dilution was performed using 0.85% saline, with a gradient range of 10^1^–10^6^ tachyzoites/ml.

Intraperitoneal injections were performed at these pre-determined gradient concentrations, with 10 C57BL/6 mice per concentration gradient and 1 ml injected per mouse. After infection, mice were monitored daily for clinical symptoms throughout the observation period. At 60 days post-infection (60 dpi), mice were euthanized, and brain cysts were counted by microscopic examination of brain homogenates.

### Genotyping and construction of phylogenetic network of *T. gondii*

Following the extraction of DNA from *T. gondii* tachyzoites, the genotypes of the isolated strains were identified by PCR–restriction fragment length polymorphism (PCR–RFLP) technology following the method of Su et al. [21]. Ten restriction sites were selected, including SAG1, SAG2 (5′ SAG2, 3′ SAG2, alt. SAG2), SAG3, BTUB, GRA6, c22-8, c29-2, L358, PK1 and Apico. All experimental batches were compared with reference strains of *T. gondii* (GT1, PTG, CTG, MAS, TgCgCa1, TgCatBr5, TgCatBr64 and TgRsCr1), and negative and blank controls were also set up.

### Quantitative real-time PCR detection

DNA was extracted from the blood of *T. gondii*-positive mice using a DNA extraction kit (DP304-03; Tiangen Biotech Co., Beijing, China). Primers and probes were designed based on the *T. gondii* B1 gene sequence in GenBank (accession no. AF179871) (Additional file 1: Table S2).

Total RNA was extracted from mouse brain tissues using TRIzol reagent (RC101-01; Vazyme Biotech, Nanjing, China), and reverse transcription was performed using the RevertAid RT Reverse Transcription Kit (Thermo Fisher Scientific, USA, Waltham, MA, USA). Quantitative PCR real-time PCR (qPCR) was conducted using the SYBR™ Green PCR Master Mix (catalog no. 4309155; Applied Biosystems™, Thermo Fisher Scientific). Primer information is provided in Additional file 1: Table S2. β-Actin was used as the reference gene, and normalization was carried out with blank controls. Relative gene expression was calculated using the formula 2^(−△△CT)^.

### Open field test

The open field test (OFT) was used to assess anxiety behavior and spontaneous activity levels in animals (*n* = 6 per group). The experimental apparatus of the OFT consisted of a 50 × 50 × 50-cm open field, divided into a central and an edge area. Mice were placed individually in the central area, and each experiment lasted 5 min. During each experiment, a video tracking system (ANY-maze) recorded the mice's movement trajectories. The following indicators were analyzed: total distance traveled, time spent in the central area and time spent stationary. After each experiment, the apparatus was wiped with 75% alcohol and thoroughly dried to prevent animal odors from affecting the results.

### Histopathological assessment

Following collection of the intestinal tissues of mice, the tissues were fixed overnight in 4% paraformaldehyde, then sectioned into 5-μm-thick slices and stained with hematoxylin and eosin (H&E). The pathological changes in the tissues were observed under an optical microscope (Olympus, Tokyo, Japan). Intestinal pathological damage was scored based on the intestinal pathology scoring system described by Yamada et al. [22], with minor modifications. The scoring criteria were: (i) epithelial integrity, with 0 = intact epithelium without cell loss, 1 = mild epithelial shedding or superficial erosion, 2 = moderate epithelial injury with focal ulcer formation and 3 = severe epithelial destruction with extensive ulceration or necrosis; (ii) villus structure, with 0 = normal villus morphology, 1 = mild villus shortening or slight fusion, 2 = moderate villus atrophy with obvious fusion and 3 = severe villus flattening, fusion or disappearance; (iii) inflammatory cell infiltration, with 0 = no or minimal inflammatory cell infiltration, 1 = mild to moderate lymphocyte or monocyte infiltration in the lamina propria and 2 = severe inflammatory infiltration, including neutrophils extending to the submucosa; and (iv) edema and hemorrhage, with 0 = absent and 1 = mild to moderate lamina propria edema or punctate hemorrhage.

 Each parameter was evaluated independently, and the highest score within each category was recorded without summing similar lesions. All sections were scored independently by at least two blinded observers, and the final score represents the average value.

### Metagenomic sequencing and metabolomics analysis

In this study, metagenomic sequencing technology was employed to analyze the microbial communities in fecal samples. Genomic DNA was extracted from the fecal samples using the Stool DNA Extraction Kit (Tiangen Biotech) according to the manufacturer’s instructions, followed by high-throughput sequencing using the NovaSeq 6000 system with PE150 sequencing mode (Illumina, Inc., San Diego, CA, USA). The sequencing data underwent quality control to remove low-quality sequences and host DNA contamination. The cleaned data were assembled using MEGAHIT software to generate contigs, and gene prediction was performed on the assembly results. Functional annotation was achieved by comparisons with annotations in metabolomics databases (including the NR, GO and KEGG databases). Microbial community diversity was evaluated through α-diversity and β-diversity analyses (principal component analysis [PCA] and principal coordinates analysis [PCoA]), and differences in microbial community functions were explored through statistical and enrichment analyses.

Fecal samples were used for metabolite extraction. Specifically, 5-mg samples of freeze-dried feces were thawed on ice to reduce degradation, and 120 μl of methanol containing internal standards was added to each sample to extract metabolites, which were then stored at − 20 °C for 30 min. After centrifugation, the supernatant was transferred and vacuum-dried. The extracted samples were resuspended in 80% methanol and stored at − 80 °C for liquid chromatography–mass spectrometry (LC–MS) analysis. Separation by LC was performed using an ultra-performance liquid chromatography–tandem mass spectrometry (UPLC–MS/MS) system, and metabolites were detected using a high-resolution mass spectrometer. The mass spectrometry data were processed for peak extraction, retention time correction and metabolite identification using XCMS and CAMERA software. Annotation was conducted using the KEGG and HMDB metabolomics databases, and quantitative analysis was performed using metaX software.

### Metabolite quantitative analysis

An appropriate amount of fecal sample (0.5 g) was placed in a pre-prepared centrifuge tube. To extract water-soluble metabolites, 15 ml of 80% methanol solution was first added to the feces, followed by the addition of 15 ml of 100% methanol for ultrasonic extraction, which was performed at 150 W power and 40 kHz frequency for 30 min. Thehe mixture was then centrifuged at 3000 *g* for 5 min, and the supernatant was collected. The residue was resuspended in 15 ml of methanol, and the extraction process was repeated; after centrifugation, the supernatant from the second extraction was combined with that of the first one. The crude extract was diluted at a ratio of 1:10 before UPLC-MS/MS analysis and filtered through a 0.22-μm membrane. Metabolites in the fecal samples, including arachidonic acid (ARA), adipic acid, 15-hydroxyarachidonic acid, 13E-eicosatetraenoic acid, prostaglandin A1, resolvin D5, 16-dimethyl prostaglandin A1, 20-hydroxy–PGF2α, 20-DiHETrA, FAHF A, leukotrienes and leukotriene B4, were quantitatively analyzed by UPLC-MS/MS. Data acquisition and processing were performed using MultiQuant 3.0 software (SCIEX, Framingham, MA, USA). Metabolite separation was carried out using a Kinetex C18 column (100 × 4.6 mm, diameter: 2.6 μm; Phenomenex, Torrance, CA, USA). The mobile phase consisted of 65% solvent A (methanol) and 35% solvent B (water solution containing 0.1% formic acid and 2 mM ammonium formate), with a flow rate of 0.2 ml/min, column temperature maintained at 30 °C and an injection volume of 5 μl. Quantitative analysis was conducted using the multiple reaction monitoring (MRM) method in positive ionization mode.

### Plaque assay

TgRodGz1 tachyzoites (200 per well) were inoculated into 6-well plates seeded with HFF cells; ARA (10 μM) was then added to each well and the plates were incubated at 37 °C for 7 days. ARA (10 μM) has no significant inhibitory effect on cell growth [23]. Plaque assays were then performed. The plaques in HFF cells were fixed with methanol and stained with crystal violet solution, and the total number of plaques was observed. Plaque counting was performed using Photoshop software (Adobe Inc., San Jose, CA, USA).

### ARA treatment of mouse model

Five-week-old C57BL/6 mice (*n* = 30) were randomly divided into five groups: (i) TgRodGz1 infection group; (ii) TgRodGz1 + ARA treatment group; (iii) Me49 infection group; (iv) Me49 + ARA treatment group and (v) ARA control group. ARA (catalog no. HY-109590; MedChemExpress) was dissolved in sterile corn oil and orally administered at a dose of 5 mg/kg body weight, which was selected based on previously reported studies investigating the immunomodulatory effects of ARA in murine models [24, 25]. An antibiotic mixture (Abx) contained vancomycin 0.5 g/l, ampicillin 1 g/l, neomycin 1 g/l, metronidazole 1 g/l in 100 ml of drinking water. In the TgRodGz1 and Me49 infection groups, each group was gavaged with 20 *T. gondii* cysts, with a mean (± standard deviation [SD] diameter of 40 ± 5.7 µm.

### Enzyme-linked immunosorbent assay

The related cytokines in the peripheral blood of mice were measured using the appropriate enzyme-linked immunosorbent assay (ELISA). Blood was collected from the orbital vein of the mice, left to stand at room temperature for 30 min and then stored overnight at 4 °C. The samples were subsequently centrifuged at 1000 *g* for 20 min, and the supernatant was collected and stored at − 20 °C. Serum immune factors, including tumor necrosis factor-alpha (TNF-α), interferon-gamma (IFN-γ) and interleukin (IL)-6, IL-1β and IL-10, were quantified using ELISA kits (Cusabio [Wuhan Cusabio Biotech Co., Ltd.], Wuhan, China). The absorbance (OD value) was measured at 450 nm with an ELISA reader.

### Immunofluorescence analysis

The embedded mouse brain tissue was sectioned into 5-μm-thick slices for immunofluorescence analysis. Prior to staining, brain tissue was fixed in 4% paraformaldehyde and washed with phosphate-buffered saline (PBS). The slices were then permeabilized with 0.2% Triton X-100 in PBS for 15 min and blocked with 5–10% normal serum for 30 min. Diluted primary antibody against Iba1 (1:500; catalog no. 10904-1-AP; Proteintech, Wuhan, China) was added and the solution incubated overnight at 4 °C. The slices were subsequently washed three times with PBS for 5 min each time. Goat Anti-Rabbit IgG H&L (Cy3®) (1:200; catalog no. ab97035; Abcam, Cambridge, UK) was added and the solution incubated for 1 h, followed by nuclear staining with DAPI (1:5000). Finally, the slices were washed three times with PBS and mounted with anti-fade mounting medium. Fluorescence images were captured using an Olympus BX53 fluorescence microscope (Olympus). Quantitative analysis of fluorescence signals and Iba1-positive cells was performed using ImageJ software (NIH, Bethesda, USA).

### Statistical analysis

All data are expressed as the mean ±  SD. When the data followed a normal distribution with equal variance, the significance of differences between two groups was analyzed using the t-test. One-way analysis of variance (ANOVA) was used to analyze the significance of differences between three or more groups. A two-tailed α of 0.05 was used as the threshold for statistical significance, with *P* < 0.05 considered to be statistically significant.

## Results

### Wild rodent *T. gondii* isolation and pathogenicity analysis

A novel strain of *T. gondii*, designated TgRodGz1, was isolated from wild rodents captured in Guangdong Province (Additional file 1: Table S1). PCR-positive tissues were homogenized and intraperitoneally inoculated into C57BL/6 mice for serial passage. Actively replicating tachyzoites were subsequently collected from the peritoneal fluid for downstream analyses.

**Fig. 1:**
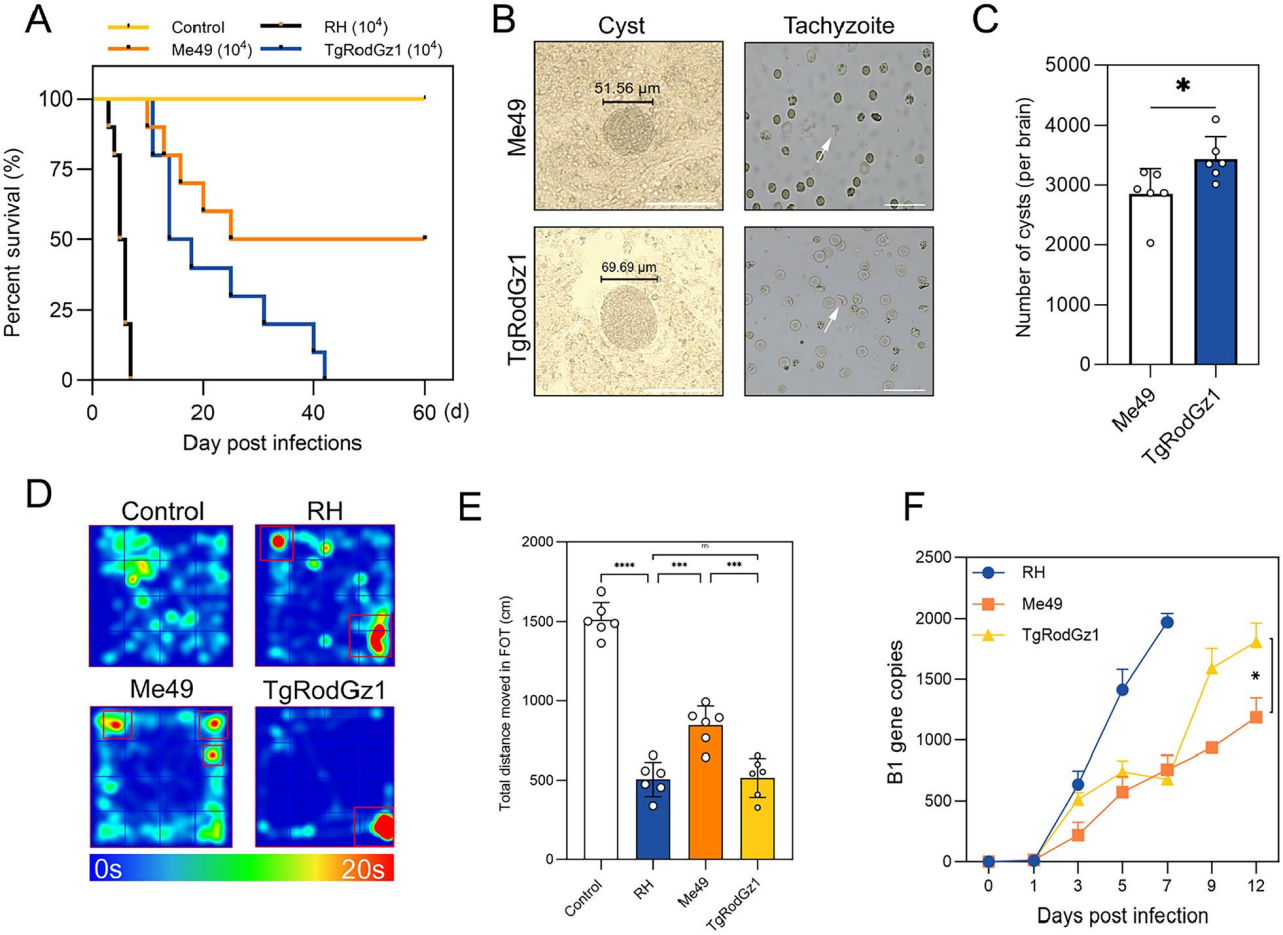
Pathogenicity analysis of*Toxoplasma gondii* TgRodGz1 strain. **A** Kaplan–Meier survival curves of C57BL/6 mice inoculated intraperitoneally with 1 × 10^4^ *T. gondii* tachyzoites of the type I RH strain (blue), the type II ME49 strain (orange) or the field isolate TgRodGz1 (yellow); phosphate-buffered saline-injected animals served as controls (black) (*n* = 6, per group). **B** Representative bright-field images of brain cysts (left) and extracellular tachyzoites (right) produced by strains ME49 or TgRodGz1; numbers indicate mean cyst diameter. Scale bar: 50/100 µm. **C** Quantification of cerebral cyst burden at 35 days post-infection. Each open circle represents one mouse (*n* = 6, per group). **D** Heat maps of locomotor paths recorded during a 20-s open-field test (OFT) on day 9 post-infection; red scale denotes elapsed stay time > 20 s. **E** Total distance traveled in the OFT (*n* = 6, per group). **F** Parasite burden in peripheral blood, expressed as copies of the B1 gene. Asterisks indicate statistically significant differences at *P < 0.05, ***P < 0.001, ****P < 0.0001; ns, not significant

To evaluate the virulence and pathogenicity of TgRodGz1, we established an in vivo infection model in C57BL/6 mice. Kaplan–Meier survival analysis revealed that all mice infected with 10^4^ TgRodGz1 tachyzoites succumbed within 42 dpi. This mortality rate was significantly higher than that following infection with the Me49 strain, but lower than that following infection with the highly virulent RH strain (Fig. 1a). On day 30 post-infection, TgRodGz1-infected mice exhibited significantly higher brain cyst burdens compared to Me49-infected mice (Fig. 1b, c). The OFT further demonstrated that, compared with RH and Me49 infection, TgRodGz1 infection markedly reduced spontaneous locomotion and exploratory activity, as indicated by restricted movement trajectories (Fig. 1d) and a significant decrease in total distance (Fig. 1e).

In addition, qPCR analysis of the *T. gondii* B1 gene in peripheral blood revealed that TgRodGz1 replicated more rapidly in vivo than the Me49 strain, although to a lesser extent than the RH strain (Fig. 1f). Taken together, these findings suggest that the TgRodGz1 strain has a virulence level that falls between the typical type I RH and type II Me49 strains, exhibiting characteristics of moderate to high virulence.

### Genotype identification of *T. gondii*

**Fig. 2:**
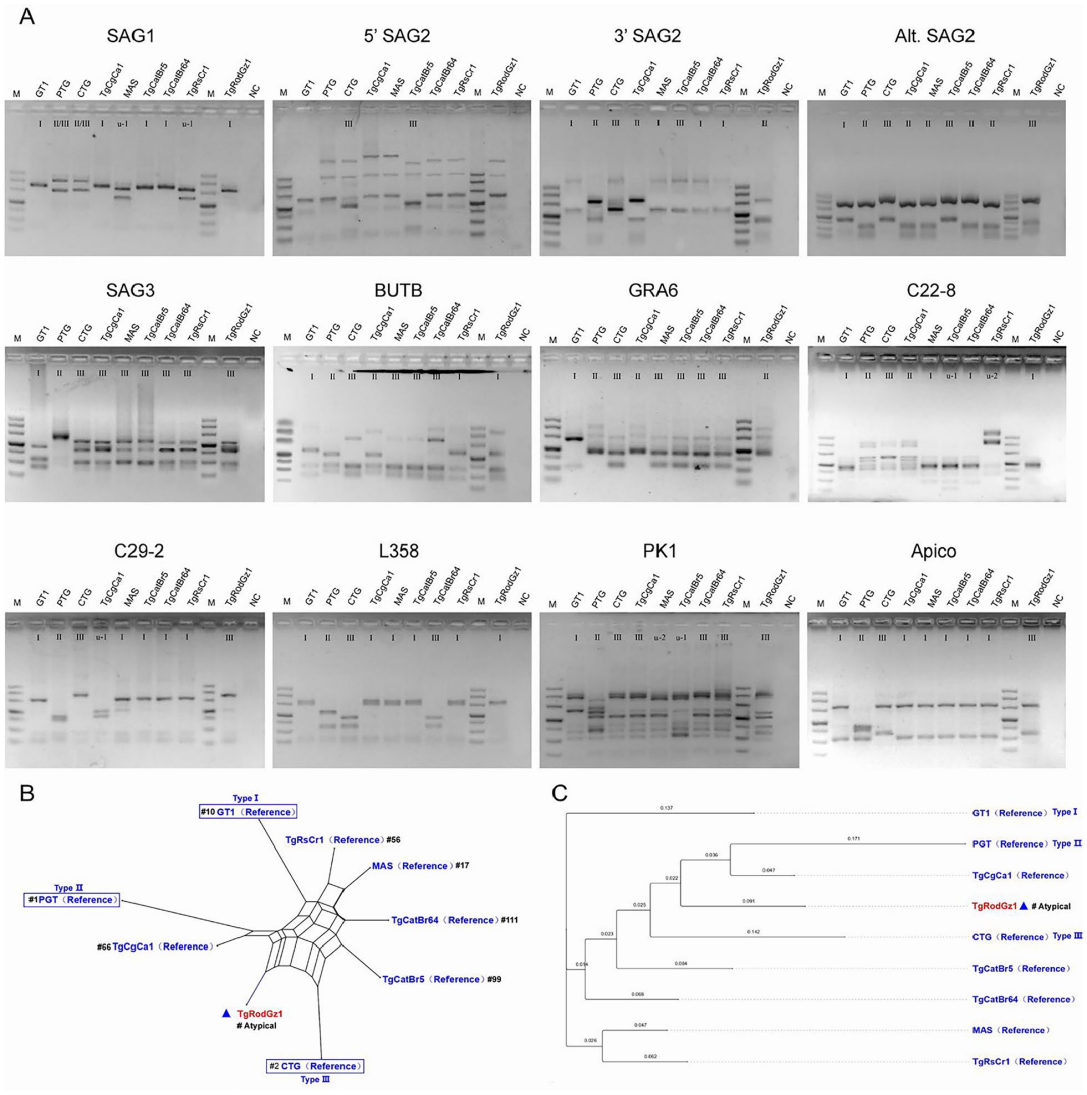
*Toxoplasma gondii* genotyping. **A** PCR–restriction fragment length polymorphism (PCR–RFLP) profiles of 12 canonical markers (SAG1, 5′-SAG2, 3′-SAG2, Alt-SAG2, SAG3, BTUB, GRA6, C22-8, C29-2, L358, PK1 and Apico) obtained for the *T. gondii* TgRodGz1 strain and reference strains. **B** Binary transformation of 12 PCR–RFLP markers was used to generate a phylogenetic network in SplitsTree4 (Neighbor-Net method). The field isolate TgRodGz1 is highlighted with a red triangle. **C** A neighbor-joining tree (1000 bootstrap replicates) built from the concatenated dataset corroborating the network topology

We used PCR–RFLP technology to genotype eight international standard reference strains, together with the TgRodGz1 isolate, using 10 genetic marker loci. The resulting genotypes were then compared with entries in the ToxoDB database (Fig. 2a; Additional file 1: Table S3). The analysis revealed that the TgRodGz1 genotype did not match any known types in the ToxoDB database or reported previously in published studies, suggesting that it represents a novel *T. gondii* genotype. The RFLP genotyping results were converted into binary data, and a phylogenetic network was constructed using the Neighbor-Net method in SplitsTree4 (Fig. 2b). A neighbor-joining (NJ) tree was also generated to confirm the genetic relationships (Fig. 2c). The results indicated that the genetic profile of TgRodGz1 differs from that of any known *T. gondii* reference strain. Phylogenetic network analysis revealed that TgRodGz1 shares greater topological similarity with the type II clonal lineage (#1, PTG), suggesting that it may belong to a subgroup within this type. In contrast, TgPgGd1 exhibited clear genetic differences. The NJ tree supported these findings, showing a branch distance of 0.091 between TgRodGz1 and the type II strain (#1, PTG), indicating a relatively close genetic relationship.

### TgRodGz1 infection induces severe intestinal injury and barrier disruption

**Fig. 3:**
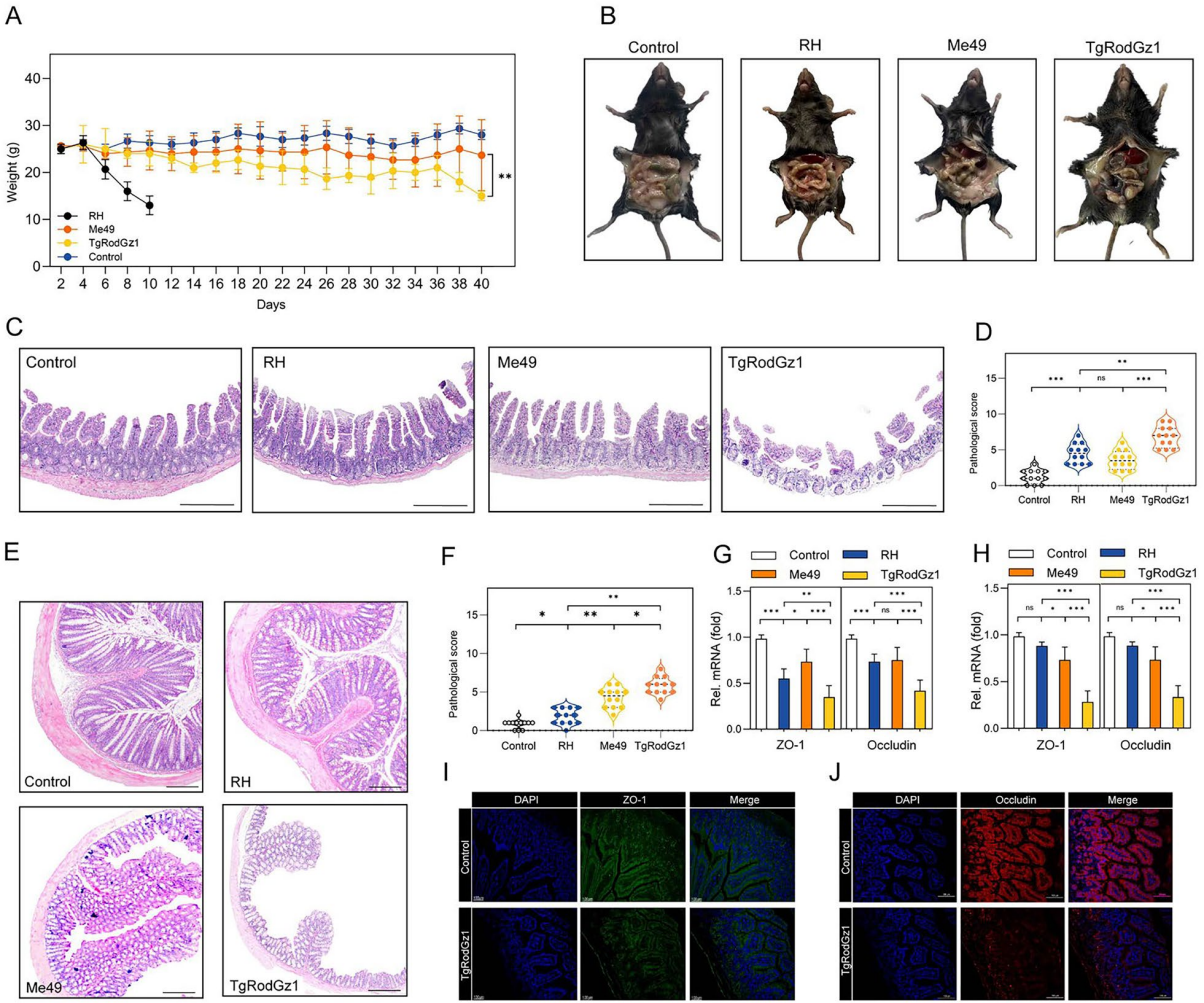
*Toxoplasma gondii* strain TgRodGz1 infection disrupts intestinal architecture and tight-junction integrity in mice. **A** Body-weight of C57BL/6 mice inoculated intraperitoneally with *T. gondii* strains RH, ME49 or TgRodGz1 tachyzoites (1 × 10^4^); phosphate-buffered saline-injected mice served as controls (*n* = 6). **B** Gross examination showing intestines in *T. gondii*-infected RH, ME49, TgRodGz1 strains and the control. **C** Hematoxylin/eosin staining of small intestines. Bar: 100 μm. **D** Small intestine pathological score (*n* = 12, per group). **E** Hematoxylin/eosin staining of colon. Bar: 100 μm. **F** Large intestine pathological score (*n* = 12). **g**, **h** Relative messenger RNA expression (quantitative/real-time PCR) of tight-junction genes ZO-1 and Occludin in the small (**G**) and large (**H**) intestine, normalized to *β*-actin. **I**, **J** Immunofluorescence staining of ZO-1 (green,** i**) and Occludin (red,** j**) with DAPI counter-stain (blue). Bar: 100 μm. Asterisks indicate statistically significant differences at *P < 0.05, **P < 0.01 ***P < 0.001; ns, not significant

To compare the intestinal pathogenicity of different *T. gondii* strains, we established mice infection models using the TgRodGz1, RH, and Me49 strains and subsequently systematically assessed changes in body weight, intestinal pathology and barrier function. All three *T. gondii* strains induced weight loss in infected mice, with the TgRodGz1-infected group exhibiting a significantly greater loss of body weight than the Me49-infected group. All mice in the RH group succumbed by day 10 post-infection (Fig. 3a).

Gross examination at day 10 post-infection revealed varying degrees of intestinal damage. RH infection caused marked mesenteric hyperemia, while Me49 infection resulted in only mild congestion. In contrast, TgRodGz1 infection induced relatively more severe intestinal pathology, including hemorrhage in both the small and large intestines, lumen dilation and bloody stools (Fig. 3b). These findings suggest that the *T. gondii* TgRodGz1 strain exhibits more pronounced intestinal pathogenic effects. H&E staining further corroborated these observations, showing that TgRodGz1 infection led to focal epithelial erosion, villus shedding, lamina propria detachment, inflammatory cell infiltration and disruption of crypt structure in the small intestine (Fig. 3c). Histopathological scoring revealed that intestinal damage was significantly more severe in the TgRodGz1-infected group than in the RH- and Me49-infected groups (Fig. 3d). In the colon, both TgRodGz1 and Me49 infections caused villus atrophy, inflammatory infiltration and lamina propria damage (Fig. 3e). These lesions were more pronounced in the TgRodGz1-infected group than in the other two groups (Fig. 3f). To assess intestinal barrier integrity, we measured messenger RNA (mRNA) levels of the tight junction proteins ZO-1 and Occludin. The expression of both genes was significantly lower in the TgRodGz1-infected group than in the RH- and Me49-infected groups (Fig. 3g, h). Additionally, immunofluorescence staining (Fig. 3i, j) confirmed a marked reduction in ZO-1 and Occludin expression in the TgRodGz1-infected group, indicating impaired intestinal barrier function. Together, these results show that infection with the TgRodGz1 isolate was associated with more pronounced intestinal tissue damage and barrier disruption compared with infection with the RH and Me49 reference strains.

### TgRodGz1 infection promotes an increase in the abundance of harmful bacteria in the gut microbiota of mice

**Fig. 4:**
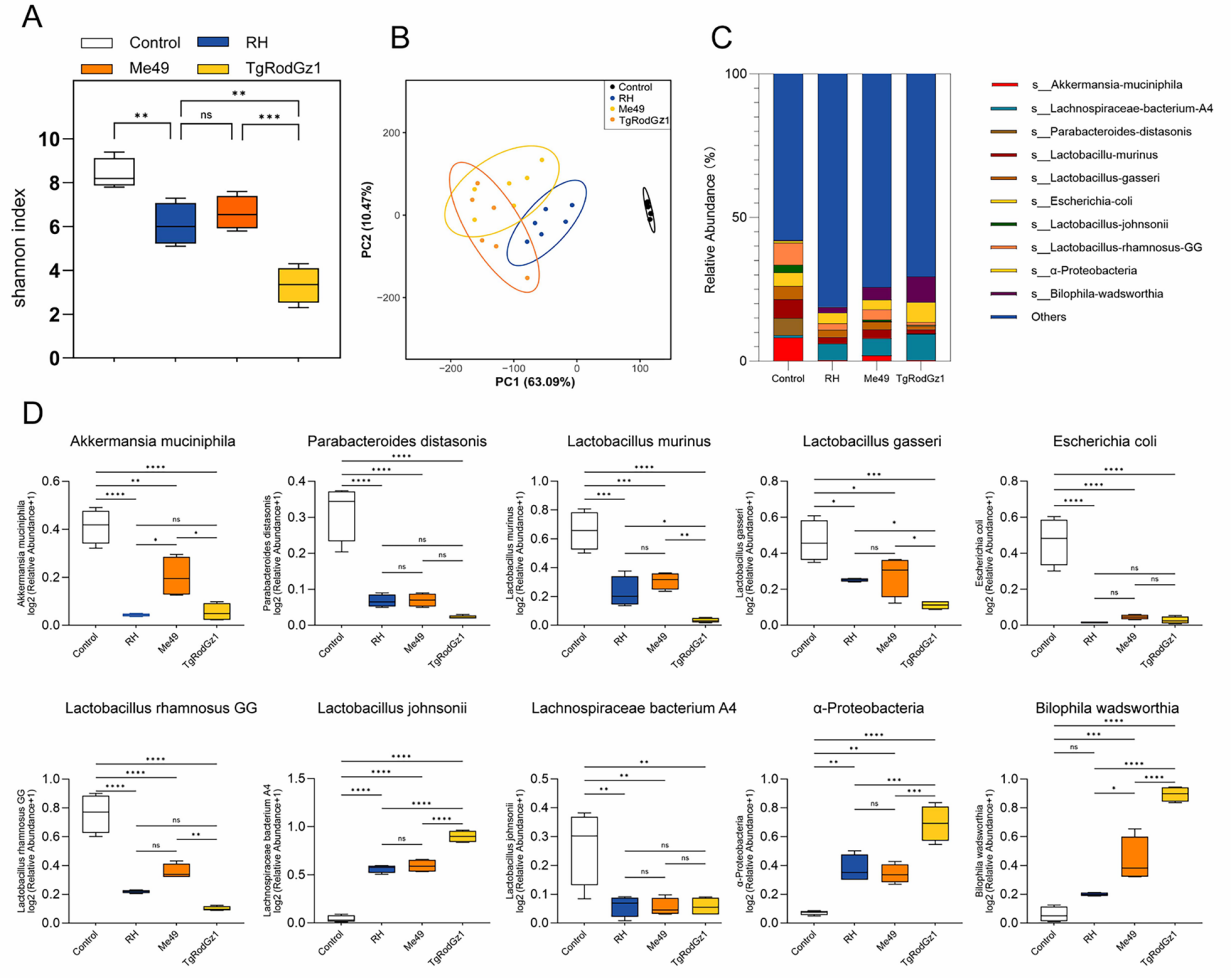
Microbial community analysis in *Toxoplasma gondii*-infected mice. **A** Boxplot of Shannon diversity index for fecal microbiota composition of the control and *T. gondii* strains RH-, Me49- and TgRodGz1-infected mice. **B** Principal coordinate analysis (PCoA) of fecal microbial composition based on Bray–curtis dissimilarity. Each data point represents a sample. **C** Stacked bar chart of relative abundance of major bacterial genera in the fecal microbiota of mice from each experimental group. **D** Boxplots of relative abundance for specific microbial taxa. Data show log-transformed relative abundance of *Akkermansia muciniphila*, *Parabacteroides distasonis, Lactobacillus murinus, Lactobacillus gasseri, Escherichia coli, Lactobacillus rhamnosus GG, Lactobacillus johnsonii, Lachnospiraceae bacterium A4, α-Proteobacteria and Bilophila wadsworthia* in control and RH-, Me49- and TgRodGz1-infected mice. Statistical comparisons are indicated for each taxon. Asterisks indicate statistically significant differences at *P < 0.05, **P < 0.01 ***P < 0.001, ****P < 0.001; ns, not significant

Dysbiosis of the gut microbiota has been recognized as a major contributor to various intestinal diseases [22]. In this study, we examined the effects of *T. gondii* infection, specifically infection with the TgRodGz1 strain, on gut microbiota composition and metabolites in mice. The Shannon index (Fig. 4a) revealed that *T. gondii* infection induced alterations in the gut microbiota, with the TgRodGz1-infected group exhibiting significantly lower microbial diversity compared with the RH-infected, Me49-infected and control groups. This finding suggests that TgRodGz1 infection exacerbates gut microbiota dysbiosis. PCoA based on Bray–Curtis distances (Fig. 4a) further demonstrated a distinct separation between the *T. gondii* infection group and the control group, supporting the hypothesis that *T. gondii* infection disrupts the gut microbiota structure. Moreover, relative abundance analysis showed significant differences in microbiota composition across the RH-, Me49- and TgRodGz1-infected groups (Fig. 4c). At the phylum level, *T. gondii* infection led to an increased dominance of Bacteroidetes, Firmicutes and Proteobacteria in the gut.

At the species level, all three *T. gondii* strains resulted in a reduction of beneficial bacteria, including *Akkermansia muciniphila*, *Parabacteroides distasonis*, *Lactobacillus murinus*, *Lactobacillus gasseri*, *Escherichia coli*, *Lactobacillus rhamnosus GG* and *Lactobacillus johnsonii.* Conversely, harmful bacteria, such as *Lachnospiraceae bacterium A4*, *α-Proteobacteria* and *Bilophila wadsworthia* ,increased in abundance in the RH-, Me49- and TgRodGz1-infected mice (Fig. 4d). Notably, the TgRodGz1 infection group displayed a significant increase in harmful bacteria, especially *α-Proteobacteria* and *Bilophila wadsworthia*, compared to the RH- and Me49-infected mice, suggesting that the *T. gondii* infection induces a typical intestinal flora imbalance, which may be linked to increased intestinal inflammation and compromised barrier function [26].

**Fig. 5:**
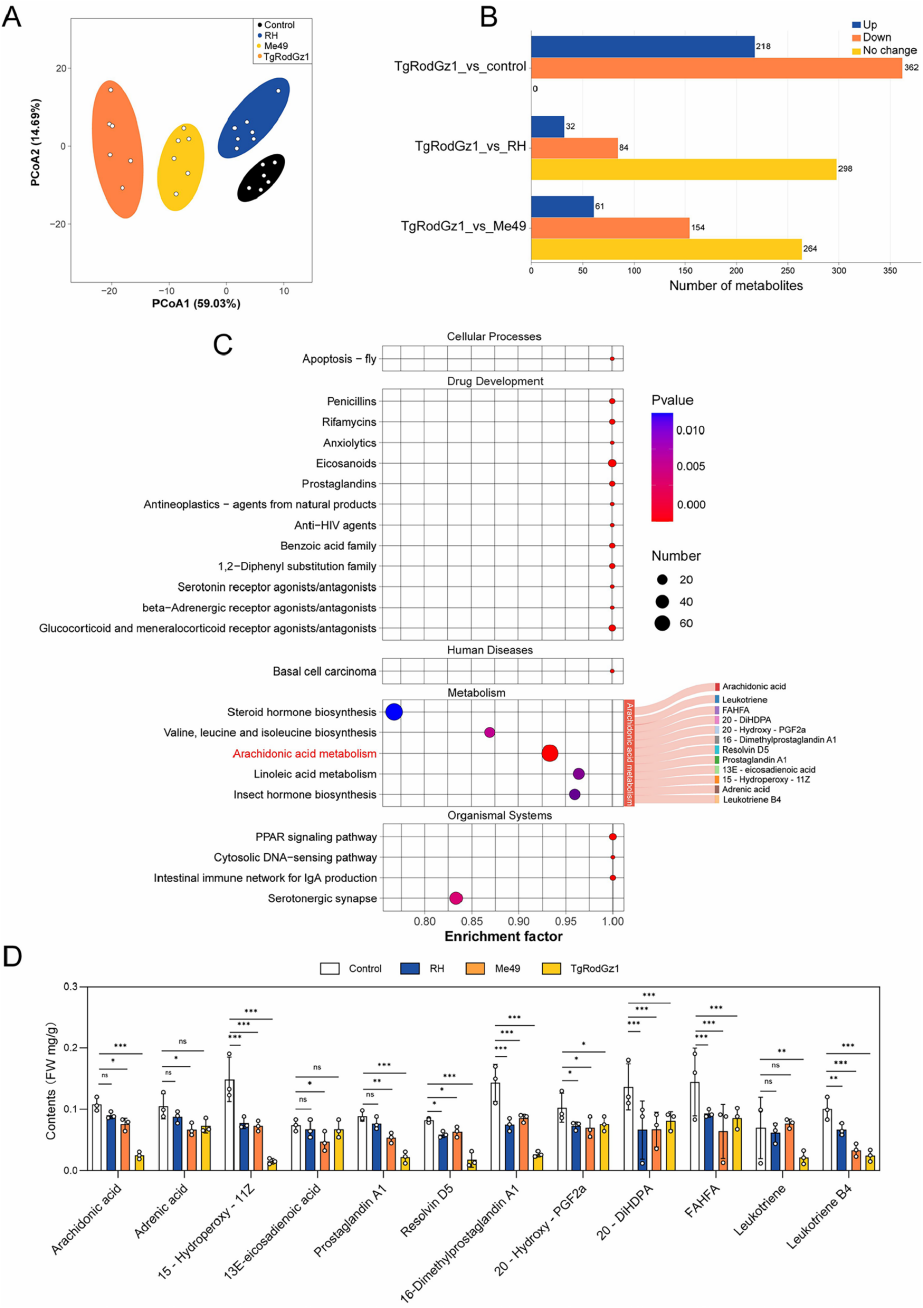
*Toxoplasma gondii*-infected metabolome analysis. **A** Principal component analysis (PCA) of untargeted liquid chromatography–tandem mass spectrometry data (LC–MS/MS) (PC1 = 59.03%, PC2 = 14.65%) distinguishes the metabolic profiles of the control and RH-, Me49- and TgRodGz1-infected mice (*n* = 6, per group). **B** Summary of differentially abundant metabolites (|fold change| > 1.2, *P* < 0.05) for *T. gondii* strain TgRodGz1-infected mice versus the control and *T. gondii* strain strains RH- and Me49-infected mice. **C** KEGG pathway enrichment of TgRodGz1-specific metabolites. Bubble size denotes the number of mapped metabolites, and the *x*-axis shows the enrichment factor. Pathways linked to arachidonic acid metabolism are markedly enriched. **D** LC–MS/MS quantification of representative lipid mediators. Asterisks indicate statistically significant differences at *P < 0.05, **P < 0.01 ***P < 0.001; ns, not significant

Gut microbiome-associated metabolites have been shown to regulate the onset and progression of gut-related diseases [27]. In this study, we found that infection with the TgRodGz1 strain resulted in significant metabolic differences compared with infection with the standard strains. PCoA (Fig. 5a) revealed that the TgRodGz1 group was distinctly separated from the control, RH and Me49 groups, indicating alteration in the metabolic profile.

Metabolite profiling further indicated that, compared to the control group, the TgRodGz1 group had 218 upregulated metabolites and 352 downregulated metabolites. In comparison with the RH group, 32 metabolites were upregulated and 84 downregulated in the TgRodGz1 group; and when compared with the Me49 group, 61 metabolites were upregulated and 154 downregulated in the TgRodGz1 group (Fig. 5b). These results highlight that TgRodGz1 infection induces notable metabolic differences compared to the control and RH- and Me49-infected mice.

Furthermore, KEGG pathway enrichment analysis showed that metabolites from the TgRodGz1, RH, and Me49 groups were enriched in pathways such as steroid hormone biosynthesis; valine, leucine and isoleucine biosynthesis; ARA metabolism; linoleic acid metabolism; and insect hormone biosynthesis. Among these, the ARA metabolism pathway exhibited the most significant enrichment, with both higher enrichment factors and statistical significance compared to other pathways (Fig. 5c).

To further assess the changes in metabolite levels, we conducted high-performance liquid chromatography (HPLC) analysis of key metabolites in the ARA metabolism pathway (Fig. 5d). Compared to the control group, the TgRodGz1 group exhibited a significant decrease in levels of ARA, leukotrienes (Leukotriene, Leukotriene B4), prostaglandins (Prostaglandin A1, 16-Dimethylprostaglandin A1) and Resolvin D5. This decrease was more pronounced than in the RH and Me49 groups. These results suggest that TgRodGz1 infection may significantly inhibit the synthesis of key pro-inflammatory lipid mediators within the ARA metabolism pathway [28, 29]. Such inhibition could exacerbate gut immune responses and inflammation, ultimately leading to severe pathological damage.

### ARA alleviates gut–brain axis damage induced by *T. gondii* infection

**Fig. 6:**
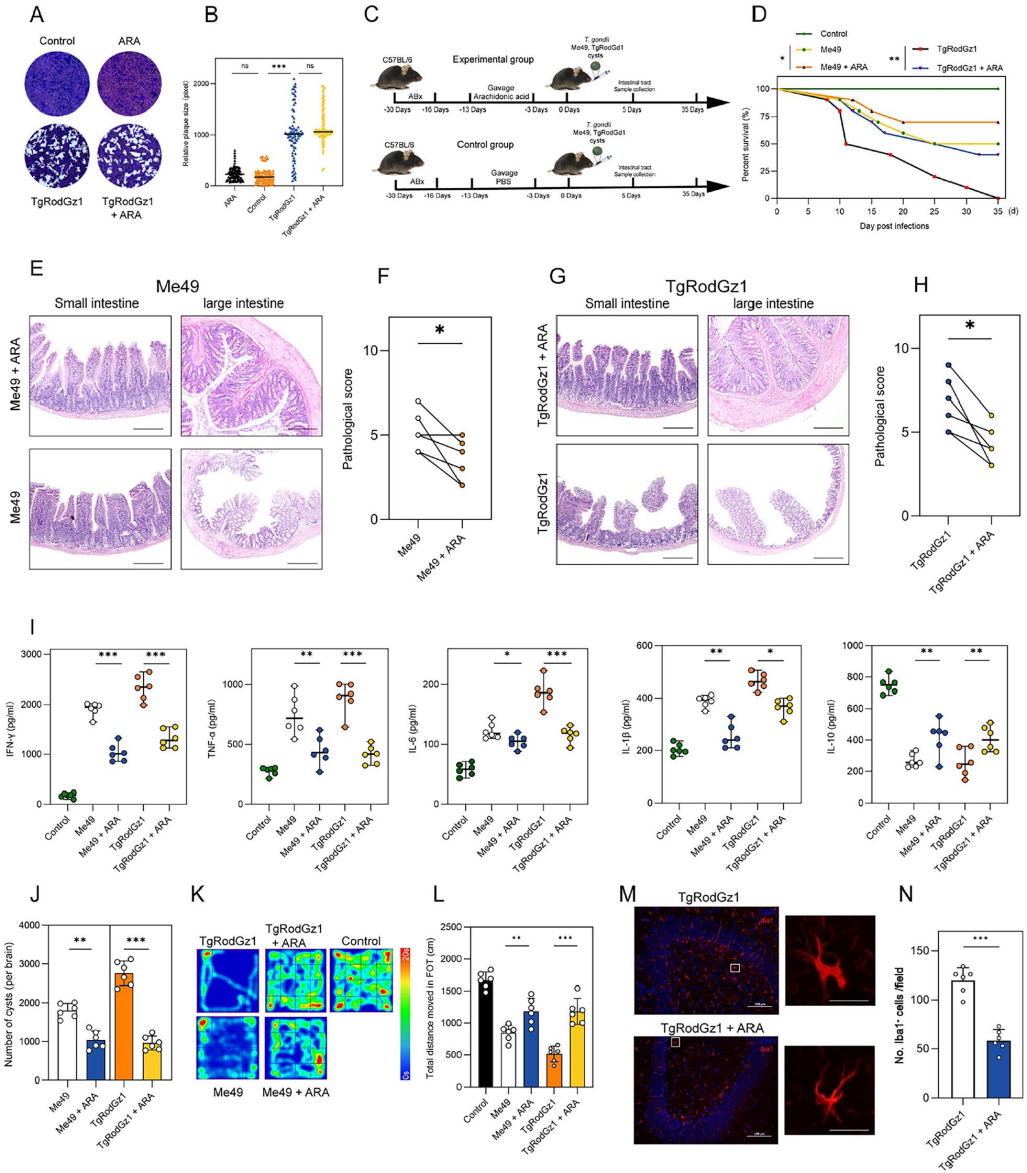
Arachidonic acid mitigates *Toxoplasma gondii*-induced damage along the gut–brain axis. **A** Crystal-violet plaque assays in human foreskin fibroblast (HFF) cells. **B** Quantification of plaque areas (*n* = 80 plaques per group). **C** Experimental timeline. **D** Kaplan–Meier survival curves (*n* = 10 per group); differences assessed by log-rank test. **e**–**h** Representative hematoxylin/eosin-stained sections and pathology scores of small (**E**, **F**) and large intestines (**G**, **H**) in*T. gondii* strains TgRodGz1- and Me49-infected mice. Bars: 100 µm. **I** Serum cytokine concentrations of IFN-γ, TNF-α, IL-6, IL-1β and IL-10 (ELISA, *n* = 6). **J** Brain cyst burden determined by cyst staining (*n* = 6 per group). **k**, **l** Open field test: Time heatmaps (**K**) and total distance traveled (**l**). **m**, **n** Iba-1 immunofluorescence, with insets showing ramified microglia (scale bars: 50 µm) (**m**) and quantification of Iba-1⁺ cells per field (*n* = 6; paired *t*-test) (**n**). Asterisks indicate statistically significant differences at *P < 0.05, **P < 0.01 ***P < 0.001; ns, not significant. ARA, Arachidonic acid; ELISA, enzyme-linked immunosorbent assay; IFN, interferon; IL, interleukin; TNF, tumor necrosis factor

Arachidonic acid is crucial for maintaining intestinal function [30]. We hypothesized that TgRodGz1 infection may aggravate intestinal immune responses and inflammation by suppressing the synthesis of ARA and its derivatives, thereby exacerbating pathological damage. To evaluate this hypothesis, we first carried out in vitro experiments to investigate whether ARA could directly inhibit the replication of *T. gondii*. Plaque assays were performed using *T. gondii*, and the results showed that the exogenous addition of ARA did not significantly affect *T. gondii* replication (Fig. 6a, b). These results indicate that ARA does not directly inhibit parasite replication under in vitro conditions and suggest that its effects may occur through host-related mechanisms in vivo*.*To eliminate the interference of other intestinal microorganisms, mice were pretreated with a cocktail of four antibiotics (vancomycin 0.5 g/l, ampicillin 1 g/l, neomycin 1 g/l, metronidazole 1 g/l) [31]. After 14 days of antibiotic pretreatment, the experimental group received ARA via gavage, while the control group was gavaged with PBS for a duration of 10 days. Subsequently, the mice were gavaged with cysts from both the TgRodGz1 isolated strain and the Me49 standard strain to assess whether ARA confers broad-spectrum resistance against *T. gondii* in the intestine (Fig. 6C).

We observed that mice infected with TgRodGz1 began to die on day 8, with a mortality rate reaching 100% by 35 dpi; in contrast, mice in the ARA treatment group (TgRodGz1 + ARA) started to die on day 12, and the mortality rate was 60% by 35 dpi, indicating that ARA treatment significantly delayed mortality associated with *T. gondii* infection. Furthermore, mice infected with the Me49 strain began to die on day 10, with a mortality rate of 50% by 35 dpi; however, in the ARA treatment group (Me49 + ARA), the mortality rate decreased to 30%, further supporting the potential protective effect of ARA in reducing mortality caused by different *T. gondii* strains (Fig. 6d).

To further evaluate the effect of ARA treatment on intestinal pathological changes, we observed the pathological features of the small and large intestines in Me49- and TgRodGz1-infected mice using H&E staining. The results showed that ARA treatment significantly improved the intestinal lesions induced by the Me49 and TgRodGz1 strains, including villus damage, inflammatory cell infiltration and detachment of the lamina propria (Fig. 6e–h). Notably, in terms of the structural integrity of both the small and large intestinal tissues, the ARA treatment group exhibited better intestinal protection, indicating its role in alleviating intestinal damage caused by *T. gondii* infection.

The levels of immune factors in the serum of TgRodGz1- and Me49-infected mice were further assessed, including IFN-γ, TNF-α, IL-6, IL-1β and IL-10 (Fig. 6i). We found that the levels of pro-inflammatory factors (IFN-γ, TNF-α, IL-6, IL-1β) were significantly elevated in the TgRodGz1-infected group, and were higher than those in both the Me49-infected and control groups, indicating that TgRodGz1 infection induced a strong immune activation and inflammatory response. In addition, the anti-inflammatory factor IL-10 was significantly lower in the TgRodGz1-infected group than in the other groups, suggesting that the inflammatory response triggered by the infection was not effectively regulated by the immune system. However, ARA treatment significantly reduced the levels of pro-inflammatory factors in TgRodGz1-infected mice and notably increased IL-10 levels. Similar patterns were observed in the Me49-infected mice. These findings suggest that ARA treatment can effectively inhibit the excessive immune activation caused by *T. gondii* infection, improve immune regulation, and exert a significant anti-inflammatory effect.

We further evaluated the number of *T. gondii* cysts in the brain, exploratory behavior and glial response in TgRodGz1- and Me49-infected mice to assess the effects of ARA treatment. As shown in Fig. 6j, ARA treatment significantly reduced the cyst burden in both TgRodGz1- and Me49-infected mice. The results of the OFT demonstrated that ARA treatment significantly increased the total movement distance in both TgRodGz1- and Me49-infected mice, alleviating the motor impairments and anxiety-like behavior induced by *T. gondii* infection (Fig. 6l, l). Furthermore, Iba1 staining revealed that ARA treatment significantly inhibited microglial proliferation in the brain induced by TgRodGz1 infection, suggesting that ARA effectively modulates the neuroinflammatory response triggered by *T. gondii* infection (Fig. 6m, n). Overall, these findings suggest that ARA treatment provides protective effects against *T. gondii*-induced intestinal and microglia cells injuries.

## Discussion

The virulence of *T. gondii* is influenced by its genetic background, the host immune response and environmental adaptability. Significant differences in virulence have been observed between wild-type strains and reference strains [32]. Standard laboratory strains are typically maintained through long-term in vitro passage, which may result in a relatively homogeneous genetic background and, in some cases, reduced virulence [6]. In contrast, wild isolates often retain greater environmental adaptability and may display a broader range of virulence characteristics, which could contribute to their persistence in natural hosts [33, 34].

In this study, we isolated a wild-rodent-derived *T. gondii* isolate, which we designated TgRodGz1, and compared its pathogenic characteristics with those of the reference type I strain (RH) and type II strain (Me49). The results indicated that TgRodGz1 exhibits an intermediate virulence phenotype, positioned between the Me49 (type II) and RH (type I) strains. Notably, TgRodGz1-infected mice were found to have a significantly greater number of brain cysts than Me49-infected mice, which is consistent with the observation that wild-type strains tend to display enhanced adaptability and virulence [35]. These findings further suggest that *T. gondii* strains with different genetic backgrounds may induce distinct host responses during infection.

In the present study, TgRodGz1 infection was associated with marked intestinal pathology, including hemorrhage, villus atrophy, inflammatory cell infiltration and reduced expression of tight junction proteins such as ZO-1 and Occludin, compared with infection with the RH and Me49 strains.

These pathological alterations suggest that TgRodGz1 may have a greater capacity to disrupt the intestinal barrier and trigger induce local inflammatory responses. Previous studies have shown that *T. gondii* infection can compromise the intestinal epithelial barrier and increase intestinal permeability, which may facilitate parasite persistence and transmission [12, 16]. Our results are consistent with these observations and indicate that TgRodGz1 infection is associated with pronounced intestinal pathology in the mouse model. During the acute phase of* T. gondii* infection, intestinal damage is often accompanied by alterations in gut microbiota composition and disturbances in host neuroimmune homeostasis [36]. Based on these findings, it is possible that TgRodGz1 may contribute to intestinal injury through related host–parasite interaction mechanisms.

Metagenomic analysis showed that TgRodGz1 infection was associated with marked alterations in the gut microbiota of mice. The relative abundance of several probiotic species, including *Akkermansia muciniphila*, *Parabacteroides distasonis*, *Lactobacillus murinus*, *Lactobacillus gasseri*, *Escherichia coli*, *Lactobacillus rhamnosus GG* and *Lactobacillus johnsonii*, was markedly reduced. In contrast, the levels of potentially pathogenic bacteria, such as *Lachnospiraceae bacterium A4*, α-*Proteobacteria* and *Bilophila wadsworthia*, were significantly increased. Alterations in the abundance of beneficial commensal bacteria may influence host immune homeostasis and contribute to intestinal inflammation [19]. Among these taxa, *Akkermansia muciniphila* has been reported to play a crucial role in maintaining intestinal barrier integrity and modulating host immune responses [37]. Reduced abundance of *Akkermansia muciniphila* has been linked to various metabolic disorders and immune dysfunctions [38]. Similarly, commensal bacteria, such as *Parabacteroides distasonis* and *Lactobacillus murinus*, have been reported to contribute to intestinal barrier maintenance, partly through the production of short-chain fatty acids (SCFAs) and modulation of host immune responses [39, 40]. Therefore, the significant reduction in probiotics may weaken intestinal immune defense, thereby facilitating the aggravation of intestinal pathological damage by *T. gondii* infection.

In addition, the increased abundance of harmful bacteria may contribute to the alterations in gut microbiota imbalance during TgRodGz1 infection. Members of the *Lachnospiraceae* family have been reported to be associated with intestinal inflammation, and increased abundance of these taxa has been observed in inflammatory bowel diseases, such as ulcerative colitis and Crohn’s disease [41, 42]. Another bacterium of interest, *Bilophila wadsworthia*, produces the metabolite hydrogen sulfide, which has been reported to influence intestinal microbial balance and host epithelial function. Hydrogen sulfide has been shown to exert cytotoxic effects on intestinal epithelial cells and may impair epithelial barrier integrity, partly through the induction of pro-inflammatory cytokines such as IL-6 and TNF-α [26, 43].

Increased abundance of α-*Proteobacteria* is associated with enhanced inflammatory responses. Members of this bacterial group can produce endotoxins, which may enter the bloodstream when intestinal barrier integrity is compromised and contribute to systemic inflammatory responses [44, 45]. Based on these observations, TgRodGz1 infection may be associated with reductions in beneficial commensal bacteria and increases in potentially harmful taxa, which could contribute to intestinal inflammation and tissue damage.

Metabolomic analysis showed that intestinal metabolites in TgRodGz1-infected mice differed from those observed in the RH- and Me49-infected mice. Notably, within the ARA metabolic pathway, the levels of ARA and several of its derivatives were reduced in the intestines of TgRodGz1-infected mice. Previous studies have reported that ARA and its downstream metabolites play important roles in maintaining intestinal barrier function and integrity and regulating inflammatory responses [46]. For example, metabolites such as prostaglandin E2 (PGE2) have been reported to promote intestinal epithelial cell regeneration and enhance the expression of tight junction proteins, thereby contributing to intestinal barrier maintenance [46, 47]. In contrast, lipid mediators such as lipoxin A4 (LXA4) are recognized as pro-resolving signals in inflammation and may limit excessive neutrophil infiltration and the persistence of inflammatory responses [48].

In the present study, we observed reduced levels of ARA and its derivatives in TgRodGz1-infected mice, suggesting that T*. gondii* infection may be associated with alterations in intestinal lipid metabolism. Authors of previous studies have proposed that* T. gondii* infection may influence host lipid metabolic pathways, including phospholipase A2 activity, peroxisome proliferator-activated receptor (PPAR) signaling and fatty acid β-oxidation, which could affect host immune responses and intestinal barrier integrity, weaken the host intestinal immunity and breach the intestinal barrier [49–51]. To further explore the potential role of ARA during* T. gondii* infection, we evaluated the effects of oral ARA supplementation in mice infected with the wild rodent-derived* T. gondii* strain TgRodGz1 or the reference strain Me49. ARA supplementation was associated with reduced mortality and changes in inflammatory cytokine levels in all infected mice, including decreased levels of pro-inflammatory cytokines (IFN-γ, TNF-α, IL-6 and IL-1β) and increased IL-10 levels. ARA is a precursor of multiple bioactive lipid mediators involved in immune regulation and inflammatory responses [46, 52]. Its metabolites, including PGE2 and lipoxin A4 (LXA4), have been reported to participate in both host defense and the resolution of inflammation, thereby contributing to immune balance under certain conditions [53]. Additionally, the ARA supplementation group exhibited significantly lower intestinal pathology scores compared to the infected control group. These results suggest that ARA may partially mitigate intestinal injury caused by *T. gondii* infection by regulating the overactivated proinflammatory response and restoring anti-inflammatory regulation. Studies have shown that alpha-linolenic acid (ALA) has an alleviating effect on *T. gondii*-induced intestinal inflammation [18], indicating that lipid-mediated immunomodulatory mechanisms may play a role in host responses to parasite-induced intestinal inflammation.

In addition, alterations in ARA metabolism are linked to central inflammatory responses and increased blood–brain barrier permeability [54]. In our study, ARA supplementation was associated with reduced brain cyst burden in TgRodGz1- and Me49-infected mice and partially improved locomotor activity in the OFT. We also observed increased microglial activation (Iba1⁺ cell counts) in the brains of TgRodGz1- and Me49-infected mice, whereas ARA supplementation was associated with reduced microglial activation.

Microglia represent the primary innate immune cells of the central nervous system, and their activation can influence neuronal survival and synaptic plasticity through the release of inflammatory mediators and phagocytic activity [55]. Therefore, modulation of microglial activation by ARA may contribute to the regulation of neuroinflammatory responses and maintenance of neural microenvironment homeostasis [56]. Taken together, these observations suggest that ARA supplementation may attenuate neuroinflammatory changes associated with* T. gondii *infection, possibly through effects on parasite burden and host inflammatory responses in the brain. However, the precise mechanisms by which ARA influences host responses during* T. gondii* infection remain unclear and require further investigation, particularly with respect to its potential roles during the acute and chronic stages of infection.

## Conclusions

In summary, our study showed that infection with the rodent-derived *T. gondii* isolate TgRodGz1 was associated with pronounced intestinal barrier disruption, alterations in gut microbiota composition and neuroinflammatory changes in mice. In addition, ARA supplementation was associated with partial improvement of inflammatory cytokine balance and attenuation of intestinal pathology and microglial activation during *T. gondii* infection. These findings suggest that ARA may influence host inflammatory responses during *T. gondii* infection and could represent a potential modulatory factor in host parasite interactions.

## Supplementary Information

**Additional file 1: Table S1. **Sample sources.** Table S2.** Primers and probe.** Table S3. **PCR-RFLP genotyping.

## Data Availability

The metagenome raw sequence data reported in this paper have been deposited in the Genome Sequence Archive in National Genomics Data Center, China National Center for Bioinformation (GSA: CRA028689) that are publicly accessible at: https://ngdc.cncb.ac.cn/gsa/browse/CRA028689. Metabolomics raw data have been deposited in figshare [57] (https://figshare.com) at: 10.6084/m9.figshare.29949737) (reference number 29949737).

## References

[1] Galeh TM, Sarvi S, Hosseini SA, Daryani A. Genetic diversity of *Toxoplasma gondii* isolates from rodents in the world: a systematic review. Transbound Emerg Dis. 2022;69:943–57. 33825346 10.1111/tbed.14096

[2] du Plooy I, Mlangeni M, Christian R, Tsotetsi-Khambule AM. An African perspective on the genetic diversity of *Toxoplasma gondii*: a systematic review. Parasitology. 2023;150:551–78. 36938833 10.1017/S0031182023000252 PMC10260301

[3] Ali Awad HA, Sardjono TW, Fitri LE, Aulanni’am A, Mohamed Sharif MA. Molecular prevalence and genetic diversity of *Toxoplasma gondii* in free-range chicken in Northeastern Libya. Open Vet J. 2023;13:225–32. 37073245 10.5455/OVJ.2023.v13.i2.11 PMC10105793

[4] Shwab EK, Saraf P, Zhu XQ, Zhou DH, McFerrin BM, Ajzenberg D, et al. Human impact on the diversity and virulence of the ubiquitous zoonotic parasite *Toxoplasma gondii*. Proc Natl Acad Sci USA. 2018;115:E6956–63. 29967142 10.1073/pnas.1722202115 PMC6055184

[5] Gerhold RW, Saraf P, Chapman A, Zou X, Hickling G, Stiver WH, et al. *Toxoplasma gondii* seroprevalence and genotype diversity in select wildlife species from the southeastern United States. Parasit Vectors. 2017;10:508. 29061166 10.1186/s13071-017-2456-2 PMC5654087

[6] Sibley LD, Khan A, Ajioka JW, Rosenthal BM. Genetic diversity of *Toxoplasma gondii* in animals and humans. Philos Trans R Soc Lond B Biol Sci. 2009;364:2749–61. 19687043 10.1098/rstb.2009.0087 PMC2865090

[7] Sibley LD, Ajioka JW. Population structure of *Toxoplasma gondii*: clonal expansion driven by infrequent recombination and selective sweeps. Annu Rev Microbiol. 2008;62:329–51. 18544039 10.1146/annurev.micro.62.081307.162925

[8] Dardé ML. *Toxoplasma gondii*, “new” genotypes and virulence. Parasite. 2008;15:366–71. 18814708 10.1051/parasite/2008153366

[9] Soldati-Favre D. Molecular dissection of host cell invasion by the apicomplexans: the glideosome. Parasite. 2008;15:197–205. 18814681 10.1051/parasite/2008153197

[10] Boothroyd JC, Dubremetz JF. Kiss and spit: the dual roles of *Toxoplasma* rhoptries. Nat Rev Microbiol. 2008;6:79–88. 18059289 10.1038/nrmicro1800

[11] Shwab EK, Zhu XQ, Majumdar D, Pena HF, Gennari SM, Dubey JP, et al. Geographical patterns of *Toxoplasma gondii* genetic diversity revealed by multilocus PCR-RFLP genotyping. Parasitology. 2014;141:453–61. 24477076 10.1017/S0031182013001844

[12] Briceño MP, Nascimento LA, Nogueira NP, Barenco PV, Ferro EA, Rezende-Oliveira K, et al. *Toxoplasma gondii* infection promotes epithelial barrier dysfunction of Caco-2 cells. J Histochem Cytochem. 2016;64:459–69. 27370796 10.1369/0022155416656349 PMC4971781

[13] Jones EJ, Korcsmaros T, Carding SR. Mechanisms and pathways of *Toxoplasma gondii* transepithelial migration. Tissue Barriers. 2017;5:e1273865. 28452683 10.1080/21688370.2016.1273865 PMC5362999

[14] Yarovinsky F, Zhang D, Andersen JF, Bannenberg GL, Serhan CN, Hayden MS, et al. TLR11 activation of dendritic cells by a protozoan profilin-like protein. Science. 2005;308:1626–9. 15860593 10.1126/science.1109893

[15] Muñoz M, Heimesaat MM, Danker K, Struck D, Lohmann U, Plickert R, et al. Interleukin (IL)-23 mediates *Toxoplasma gondii*-induced immunopathology in the gut via matrixmetalloproteinase-2 and IL-22 but independent of IL-17. J Exp Med. 2009;206:3047–59. 19995958 10.1084/jem.20090900 PMC2806449

[16] Hunter CA, Sibley LD. Modulation of innate immunity by *Toxoplasma gondii* virulence effectors. Nat Rev Microbiol. 2012;10:766–78. 23070557 10.1038/nrmicro2858 PMC3689224

[17] Nascimento BB, Cartelle CT, Noviello ML, Pinheiro BV, de Almeida Vitor RW, Souza DDG, et al. Influence of indigenous microbiota on experimental toxoplasmosis in conventional and germ-free mice. Int J Exp Pathol. 2017;98:191–202. 28895246 10.1111/iep.12236 PMC5639263

[18] Yang J, Liu S, Zhao Q, Li X, Jiang K. Gut microbiota-related metabolite alpha-linolenic acid mitigates intestinal inflammation induced by oral infection with *Toxoplasma gondii*. Microbiome. 2023;11:273. 38087373 10.1186/s40168-023-01681-0 PMC10714487

[19] Belkaid Y, Hand TW. Role of the microbiota in immunity and inflammation. Cell. 2014;157:121–41. 24679531 10.1016/j.cell.2014.03.011 PMC4056765

[20] Yu Q, Yuan L, Deng J, Yang Q.* Lactobacillus *protects the integrity of intestinal epithelial barrier damaged by pathogenic bacteria. Front Cell Infect Microbiol. 2015;5:26. 25859435 10.3389/fcimb.2015.00026 PMC4373387

[21] Dubey JP, Ferreira LR, Martins J, McLeod R. Oral oocyst-induced mouse model of toxoplasmosis: effect of infection with *Toxoplasma gondii* strains of different genotypes, dose, and mouse strains (transgenic, out-bred, in-bred) on pathogenesis and mortality. Parasitology. 2012;139:1–13. 22078010 10.1017/S0031182011001673 PMC3683600

[22] Yamada M, Ohkusa T, Okayasu I. Occurrence of dysplasia and adenocarcinoma after experimental chronic ulcerative colitis in hamsters induced by dextran sulphate sodium. Gut. 1992;33:1521–7. 1333439 10.1136/gut.33.11.1521 PMC1379539

[23] Solakivi T, Kunnas T, Kärkkäinen S, Jaakkola O, Nikkari ST. Arachidonic acid increases matrix metalloproteinase 9 secretion and expression in human monocytic MonoMac 6 cells. Lipids Health Dis. 2009;8:11. 19331685 10.1186/1476-511X-8-11 PMC2667508

[24] Naito Y, Ji X, Tachibana S, Aoki S, Furuya M, Tazura Y, et al. Effects of arachidonic acid intake on inflammatory reactions in dextran sodium sulphate-induced colitis in rats. Br J Nutr. 2015;114:734–45. 26234346 10.1017/S000711451500224X

[25] Tateishi N, Kakutani S, Kawashima H, Shibata H, Morita I. Dietary supplementation of arachidonic acid increases arachidonic acid and lipoxin A_4_ contents in colon, but does not affect severity or prostaglandin E_2_ content in murine colitis model. Lipids Health Dis. 2014;13:30. 24507383 10.1186/1476-511X-13-30 PMC3928921

[26] Feng Z, Long W, Hao B, Ding D, Ma X, Zhao L, et al. A human stool-derived *Bilophila wadsworthia* strain caused systemic inflammation in specific-pathogen-free mice. Gut Pathog. 2017;9:59. 29090023 10.1186/s13099-017-0208-7 PMC5657053

[27] Cryan JF, O’Riordan KJ, Cowan CS, Sandhu KV, Bastiaanssen TF, Boehme M, et al. The microbiota–gut–brain axis. Physiol Rev. 2019;99:1877–2013. 31460832 10.1152/physrev.00018.2018

[28] Wang T, Fu X, Chen Q, Patra JK, Wang D, Wang Z, et al. Arachidonic acid metabolism and kidney inflammation. Int J Mol Sci. 2019;20. 10.3390/ijms20153683 PMC6695795 31357612

[29] Chandrasekharan JA, Marginean A, Sharma-Walia N. An insight into the role of arachidonic acid derived lipid mediators in virus associated pathogenesis and malignancies. Prostaglandins Other Lipid Mediat. 2016;126:46–54. 27450483 10.1016/j.prostaglandins.2016.07.009

[30] Wang Q, Lin Y, Sheng X, Xu J, Hou X, Li Y, et al. Arachidonic acid promotes intestinal regeneration by activating WNT signaling. Stem Cell Rep. 2020;15:374–88. 10.1016/j.stemcr.2020.06.009 PMC7419670 32649903

[31] Isselbacher KJ. The role of arachidonic acid metabolites in gastrointestinal homeostasis. Biochemical, histological and clinical gastrointestinal effects. Drugs. 1987;33:38–46. 2885170 10.2165/00003495-198700331-00007

[32] Saraf P, Shwab EK, Dubey JP, Su C. On the determination of *Toxoplasma gondii* virulence in mice. Exp Parasitol. 2017;174:25–30. 28153801 10.1016/j.exppara.2017.01.009

[33] Sibley LD, Mordue DG, Su C, Robben PM, Howe DK. Genetic approaches to studying virulence and pathogenesis in *Toxoplasma gondii*. Philos Trans R Soc Lond B Biol Sci. 2002;357:81–8. 11839185 10.1098/rstb.2001.1017 PMC1692920

[34] Li M, Mo XW, Wang L, Chen H, Luo QL, Wen HQ, et al. Phylogeny and virulence divergency analyses of *Toxoplasma gondii* isolates from China. Parasit Vectors. 2014;7:133. 24678633 10.1186/1756-3305-7-133 PMC3986613

[35] Lilue J, Müller UB, Steinfeldt T, Howard JC. Reciprocal virulence and resistance polymorphism in the relationship between *Toxoplasma gondii* and the house mouse. Elife. 2013;2:e01298. 24175088 10.7554/eLife.01298 PMC3810784

[36] French T, Steffen J, Glas A, Osbelt L, Strowig T, Schott BH, et al. Persisting microbiota and neuronal imbalance following *T. gondii* infection reliant on the infection route. Front Immunol. 2022;13:920658. 35898505 10.3389/fimmu.2022.920658 PMC9311312

[37] Everard A, Belzer C, Geurts L, Ouwerkerk JP, Druart C, Bindels LB, et al. Cross-talk between *Akkermansia muciniphila* and intestinal epithelium controls diet-induced obesity. Proc Natl Acad Sci USA. 2013;110:9066–71. 23671105 10.1073/pnas.1219451110 PMC3670398

[38] Ansaldo E, Slayden LC, Ching KL, Koch MA, Wolf NK, Plichta DR, et al. *Akkermansia muciniphila* induces intestinal adaptive immune responses during homeostasis. Science. 2019;364:1179–84. 31221858 10.1126/science.aaw7479 PMC6645389

[39] Si W, Zhao X, Li R, Li Y, Ma C, Zhao X, et al.* Lactobacillus rhamnosus* GG induces STING-dependent IL-10 in intestinal monocytes and alleviates inflammatory colitis in mice. J Clin Invest. 2025;135:e174910. 10.1172/JCI174910 PMC11785918 39895628

[40] Ji HF, Li M, Han X, Fan YT, Yang JJ, Long Y, et al. Lactobacilli-mediated regulation of the microbial–immune axis: a review of key mechanisms, influencing factors, and application prospects. Foods. 2025;14:1763. 10.3390/foods14101763 PMC12111133 40428542

[41] Sun Y, Wang X, Li L, Zhong C, Zhang Y, Yang X, et al. The role of gut microbiota in intestinal disease: from an oxidative stress perspective. Front Microbiol. 2024;15:1328324. 38419631 10.3389/fmicb.2024.1328324 PMC10899708

[42] Ho JT, Chan GC, Li JC. Systemic effects of gut microbiota and its relationship with disease and modulation. BMC Immunol. 2015;16:21. 25896342 10.1186/s12865-015-0083-2 PMC4404277

[43] Davies J, Mayer MJ, Juge N, Narbad A, Sayavedra L. *Bacteroides thetaiotaomicron* enhances H(2)S production in *Bilophila wadsworthia*. Gut Microbes. 2024;16:2431644. 39609271 10.1080/19490976.2024.2431644 PMC11610557

[44] Mohr AE, Crawford M, Jasbi P, Fessler S, Sweazea KL. Lipopolysaccharide and the gut microbiota: considering structural variation. FEBS Lett. 2022;596:849–75. 35262962 10.1002/1873-3468.14328

[45] Mohammad S, Thiemermann C. Role of metabolic endotoxemia in systemic inflammation and potential interventions. Front Immunol. 2020;11:594150. 33505393 10.3389/fimmu.2020.594150 PMC7829348

[46] Wang B, Wu L, Chen J, Dong L, Chen C, Wen Z, et al. Metabolism pathways of arachidonic acids: mechanisms and potential therapeutic targets. Signal Transduct Target Ther. 2021;6:94. 33637672 10.1038/s41392-020-00443-w PMC7910446

[47] Sun Y, Wu D, Zeng W, Chen Y, Guo M, Lu B, et al. The role of intestinal dysbacteriosis induced arachidonic acid metabolism disorder in inflammaging in atherosclerosis. Front Cell Infect Microbiol. 2021;11:618265. 33816331 10.3389/fcimb.2021.618265 PMC8012722

[48] Godson C, Guiry P, Brennan E. Lipoxin mimetics and the resolution of inflammation. Annu Rev Pharmacol Toxicol. 2023;63:429–48. 36662584 10.1146/annurev-pharmtox-051921-085407

[49] Huang Y, Zhou Y, He Z, Yang J, Gu J, Cui B, et al. Cellular senescence contributes to colonic barrier integrity impairment induced by *Toxoplasma gondii* infection. Inflammation. 2025;48:2600-12. 10.1007/s10753-024-02213-0 PMC12336079 39827329

[50] Snyder LM, Denkers EY. From initiators to effectors: roadmap through the intestine during encounter of *Toxoplasma gondii* with the mucosal immune system. Front Cell Infect Microbiol. 2020;10:614701. 33505924 10.3389/fcimb.2020.614701 PMC7829212

[51] Balsinde J, Winstead MV, Dennis EA. Phospholipase A(2) regulation of arachidonic acid mobilization. FEBS Lett. 2002;531:2–6. 12401193 10.1016/s0014-5793(02)03413-0

[52] Calder PC. Omega-3 fatty acids and inflammatory processes. Nutrients. 2010;2:355–74. 22254027 10.3390/nu2030355 PMC3257651

[53] Das UN. Essentials. Biomolecules. 2021;11:1873.

[54] Murphy EJ. Blood-brain barrier and brain fatty acid uptake: role of arachidonic acid and PGE2. J Neurochem. 2015;135:845–8. 26383055 10.1111/jnc.13289

[55] Cornell J, Salinas S, Huang HY, Zhou M. Microglia regulation of synaptic plasticity and learning and memory. Neural Regen Res. 2022;17:705–16. 34472455 10.4103/1673-5374.322423 PMC8530121

[56] Lin D, Gold A, Kaye S, Atkinson JR, Tol M, Sas A, et al. Arachidonic acid mobilization and peroxidation promote microglial dysfunction in Aβ pathology. J Neurosci. 2024;44:e0202242024. 10.1523/JNEUROSCI.0202-24.2024 PMC11293449 38866484

[57] Singh J. FigShare. J Pharmacol Pharmacother. 2011;2:138–9. 21772785 10.4103/0976-500X.81919 PMC3127351

